# Daily parathyroid hormone administration enhances bone turnover and preserves bone structure after severe immobilization‐induced bone loss

**DOI:** 10.14814/phy2.13446

**Published:** 2017-09-28

**Authors:** Lauren Harlow, Karim Sahbani, Jeffry S. Nyman, Christopher P. Cardozo, William A. Bauman, Hesham A. Tawfeek

**Affiliations:** ^1^ National Center for the Medical Consequences of Spinal Cord Injury James J. Peters Veterans Affairs Medical Center Bronx New York; ^2^ Department of Orthopaedic Surgery & Rehabilitation Center for Bone Biology Vanderbilt University Medical Center Nashville Tennessee; ^3^ Department of Biomedical Engineering Center for Bone Biology Vanderbilt University Medical Center Nashville Tennessee; ^4^ Department of Medicine The Icahn School of Medicine at Mount Sinai New York New York; ^5^ Department of Rehabilitation Medicine The Icahn School of Medicine at Mount Sinai New York New York; ^6^ Department of Pharmacologic Science The Icahn School of Medicine at Mount Sinai New York New York

**Keywords:** Bone loss, immobilization, osteoporosis, PTH, spinal cord injury

## Abstract

Immobilization, as a result of motor‐complete spinal cord injury (SCI), is associated with severe osteoporosis. Whether parathyroid hormone (PTH) administration would reduce bone loss after SCI remains unclear. Thus, female mice underwent sham or surgery to produce complete spinal cord transection. PTH (80 *μ*g/kg) or vehicle was injected subcutaneously (SC) daily starting on the day of surgery and continued for 35 days. Isolated tibias and femurs were examined by microcomputed tomography scanning (micro‐CT) and histology and serum markers of bone turnover were measured. Micro‐CT analysis of tibial metaphysis revealed that the SCI‐vehicle animals exhibited 49% reduction in fractional trabecular bone volume and 18% in trabecular thickness compared to sham‐vehicle controls. SCI‐vehicle animals also had 15% lower femoral cortical thickness and 16% higher cortical porosity than sham‐vehicle counterparts. Interestingly, PTH administration to SCI animals restored 78% of bone volume, increased connectivity to 366%, and lowered structure model index by 10% compared to sham‐vehicle animals. PTH further favorably attenuated femoral cortical bone loss to 5% and prevented the SCI‐associated cortical porosity. Histomorphometry evaluation of femurs of SCI‐vehicle animals demonstrated a marked 49% and 38% decline in osteoblast and osteoclast number, respectively, and 35% reduction in bone formation rate. In contrast, SCI‐PTH animals showed preserved osteoblast and osteoclast numbers and enhanced bone formation rate. Furthermore, SCI‐PTH animals had higher levels of bone formation and resorption markers than either SCI‐ or sham‐vehicle groups. Collectively, these findings suggest that intermittent PTH receptor activation is an effective therapeutic strategy to preserve bone integrity after severe immobilization.

## Introduction

Bone is an active organ that undergoes a complex and continuous process of remodeling. This process involves resorption of bone by osteoclasts (multinucleated cells of hematopoietic origin) followed by synthesis of new bone matrix and subsequent mineralization by osteoblasts (cells of mesenchymal origin). This remodeling process is important for maintaining calcium homeostasis and preserving skeletal mass and integrity. Thus, disorders associated with impaired or negatively imbalanced bone remodeling with excessive resorption manifest with bone loss.

Spinal cord injury (SCI) is a major trauma that affects the function of many organ systems, including that of the skeletal system (Kostovski et al. 2010). Bone loss after SCI has been attributed primarily to the loss of muscle innervation/function, disuse muscle atrophy, and the subsequent immobilization. As a consequence of this reduction in mechanical loading, most patients rapidly and dramatically lose bone shortly after injury with the eventual development of osteoporosis (Bauman and Cardozo 2015). As a result, the use of rehabilitation techniques, exercise, and electrical muscle stimulation evolved as measures to restore mechanical stimulation and reduce the bone loss of acute immobilization. Other factors that are believed to further worsen bone loss after acute injury are the dysregulation of sympathetic outflow, impairment of the local circulation, and insufficient anabolic stimulation due to testosterone deficiency in men and suppression of endogenous PTH release (Huang et al. 1997; Maimoun et al. 2006b; Qin et al. 2010; Gaspar et al. 2014). Mechanistic studies suggested that bone loss is attributed to increased osteoclast activity and number, causing an imbalance in bone remodeling by favoring bone resorption over that of bone formation (Demulder et al. 1998; Jiang et al. 2006, 2007; Reiter et al. 2007; Morse et al. 2008, 2011; Battaglino et al. 2012; Sabour et al. 2014). This notion has been further supported by the finding that bone resorption markers increase several fold after injury and remain high for months to years thereafter (Uebelhart et al. 1994; Roberts et al. 1998; Nance et al. 1999; Dauty et al. 2000; Maimoun et al. 2002, 2005, 2006a). These studies promoted the use of antiresorptive agents, such as calcitonin and bisphosphonates. However, the efficacy of this approach in treating bone loss in those who become nonambulatory after SCI remains unclear and controversial (Maimoun et al. 2006a). Furthermore, findings from our investigations strongly suggest that there is no benefit of bisphosphonates, specifically pamidronate or zoledronic acid at the knee in individuals with motor‐complete SCI (Bauman et al. 2014).

An important regulator of bone remodeling is parathyroid hormone (PTH). PTH is a single‐chain, 84‐amino acid polypeptide hormone secreted by the parathyroid glands in response to hypocalcemia to maintain homeostasis of circulating levels of calcium. The primary function of endogenous PTH is to act directly on the kidney to increase calcium reabsorption and phosphate excretion, on bone to stimulate calcium mobilization, and indirectly on the intestine by the action of vitamin D to enhance calcium absorption. Repeated transient elevation of PTH achieved by daily (intermittent) administration to animals or humans, increases trabecular bone mass and bone mineral density (BMD), and improves bone biomechanical properties. For these reasons, a regimen of daily PTH administration for a circumscribed period of time has been approved by the FDA for treating primary osteoporosis associated with a high risk of fracture (Neer et al. 2001) and osteoporosis secondary to glucocorticoid use (Saag et al. 2007).

Although many reports have indicated that PTH is beneficial in unloading‐associated bone loss (Ma et al. 1995; Halloran et al. 1997; Turner et al. 1998, 2007; Ono et al. 2007; Bruel et al. 2013; Sandberg et al. 2014), whether PTH administration could protect against SCI‐induced bone loss remains unclear. In this study, we evaluated whether stimulation of bone anabolism using intermittent PTH receptor 1 (PTHR1) activation would preserve bone structure after severe immobilization in SCI animals.

## Materials and Methods

### Reagents

Human PTH(1–34) (Teriparatide, Cat. Number H‐4835) was purchased from Bachem (Torrance, CA) and was reconstituted in sterile solution of 0.9% NaCl and 10 mmol/L acetic acid. Vehicle (0.9% NaCl/10 mmol/L acetic acid) or PTH (80 *μ*g/kg/day) was injected SC daily (except Sunday) starting on the day of surgery and continued for 35 days. A PTH dose of 40–80 *μ*g/kg/day has been commonly used in studying PTH actions in bone. Additionally, a dose of 80 *μ*g/kg/day in our previous experiments on mice exhibited an optimal bone anabolic response to PTH (Terauchi et al. 2009; Bedi et al. 2012). The great difference between the mouse and human dosage (20 *μ*g/day) is anticipated given that the metabolic rate in mice is almost 10 times higher than humans. Calcein (Cat. Number C0875) was purchased from Sigma‐Aldrich (St. Louis, MO) and reconstituted using 2% sodium bicarbonate solution. Calcein (30 mg/kg) was injected to each animal SC twice (10 and 3 days before the end of the experiment).

### Animals

All animal procedures were reviewed and approved by the Institutional Animal Care and Use Committee at James J. Peters VA Medical Center. All experiments were conducted on 24‐week‐old, female C57BL/6 mice from Charles River Laboratories (Kingston, NY). There are many reasons for using mice as an appropriate model for our SCI investigations. First, mice have been widely used as very useful models for studying bone homeostasis in health and disease. Second, the effects of PTH on bone mass have been well demonstrated in normal mice and mouse models of osteoporosis. Therefore, sufficient literature and knowledge are available that can be applied to advance our understanding of bone loss after severe immobilization. Third, since most genetic modifications are generated in mice, many genetically modified mice have become available to further determine the role of different molecules in SCI‐induced bone loss. Lastly, mouse genome and physiology are similar to that of humans making the mouse an appropriate model to study human diseases.

### Spinal cord injury surgery

Spinal cord transection was performed in the thoracic segment of the spinal cord to result in complete paralysis of the hindlimbs. Animal underwent general anesthesia using controlled isoflurane inhalation from a precision vaporizer or flowmeter.

The surgical site was prepared by wiping three times with alternating 70% ethanol and betadine. A midline incision was made over spinous processes T6–T10 and paravertebral muscles were separated from the vertebrae. The laminae and spinous processes of the T9 vertebra were removed (laminectomy) with fine scissors to expose the underlying spinal cord. The spinal cord was cut with a scalpel or microscissors and a surgical sponge was placed in the gap between the ends of the severed spinal cord. Muscle and connective tissues were closed with a 4‐0 to 5‐0 absorbable suture, and the skin was closed with monofilament nonabsorbable sutures or surgical clips or staples and tissue glue. Sutures were removed at 7–10 days under anesthesia with inhaled isofluorane. Spinal cord transection results in complete paralysis of the hindlimbs. Mice lose the ability to voluntarily micturate, requiring bladders to be manually expressed three times daily initially, then at least once daily after some automatic emptying of the bladder develops. With complete hindlimb paralysis, mice can still roam freely with their forelimbs and have full access to food and water. At the time of bladder expression, mice were provided with saline or lactated Ringers's solution (1 mL). Mice also received an injection Carprofen analgesic (subcutaneously (SC), 5 mg/kg once 1 h before surgery then once daily for 3 days after surgery and subsequently as needed) and Baytril antibiotic (SC, 2.5–5 mg/kg twice daily on the day of surgery then twice daily for 3 days after surgery in saline solution). At the time of bladder expression, a health check on each mouse was performed for any visible signs of distress such as lack of responsiveness, abnormal stool, porphyria around the eyes, dehydration, weight loss, lack of grooming, lack of mobility, pressure sores, and/or urinary tract infection. If any of these signs were noticed, the mouse was placed back on the warming pad and administered Ringer's solution, Carprofen, and/or other medications, as necessary or recommended by the attending veterinarian. Mice that did not improve after 2 days, were euthanized. After surgery, mice were placed on a warming pad and monitored continuously until fully recovered from anesthesia, then three times daily for 2 weeks, including weekends and then once daily during weekdays until the end of experiment. Animals were weighed before surgery and then twice weekly until the end of experiment. Weight and health status were recorded.

For studies investigating SCI‐induced bone loss that results primarily from muscle paralysis and mechanical unloading, spinal transection is an appropriate model. Spinal cord transection in mice provides the most complete denervation and paralysis, thus closely modeling persons with motor‐complete SCI, the form of SCI associated with the most severe bone loss. Technically, contusion SCI in mice is more difficult to reproduce and the extent of SCI may vary among animals; this will cause variable degrees of bone loss that would respond differently to treatment, thus complicating the interpretation of the data. The spinal transection approach, therefore, assures similar unloading and bone loss among animals.

### Micro‐CT measurements of cortical and trabecular bone

Animals were euthanized using isoflurane inhalation (to effect) anesthesia followed by exsanguination and cervical dislocation and femurs and tibias harvested and fixed in 10% neutral buffered formalin for 24 h and then preserved in 70% ethanol. Bones were scanned using a microcomputed tomographic (micro‐CT) instrument (VivaCT‐40, Scanco Medical AG, Bassersdorf, Switzerland). Standard nomenclature and guidelines for assessment of bone microstructure were followed, as recommended by the American Society for Bone and Mineral Research (Bouxsein et al. 2010). Cross‐sectional geometry at the femoral midshaft and trabecular bone volume fraction and microarchitecture in the secondary spongiosa of the proximal tibia were assessed. The bones were scanned at a low resolution, an energy level of 55 kVp, intensity of 145 *μ*A, and a fixed threshold of 220. The VivaCT‐40 is calibrated weekly using a phantom provided by Scanco. Scans for the cortical region were measured at the midpoint of each femur, with an isotropic pixel size of 21 *μ*m and slice thickness of 21 *μ*m, and used to calculate the average total cross‐sectional area (mm^2^), bone area (mm^2^), and marrow area (mm^2^). For midshaft analysis, the cortical shell was contoured by user‐defined threshold and iterated across the 50 slices. Tibial trabecular bone volume fraction and microarchitecture were evaluated in the secondary spongiosa, starting proximately at 0.6 mm distal to the growth plate, and extending distally 1.5 mm. Approximately 230 consecutive slices were made at 10.5 *μ*m interval at the distal end of the growth plate and extending in a proximal direction, and 180 contiguous slices were selected for analysis. The main bone parameters are TV (total volume [mm^3^]), BV (bone volume [mm^3^]), BV/TV (the relative volume of calcified tissue in the selected volume of interest (VOI), Tb.Th (the thickness of the trabecular structure), Tb.N (the number of trabeculae), Tb.Sp (trabecular separation; a measurement of the thickness of the spaces between the trabeculae, inversely proportional to the trabecular thickness), Conn. D (connectivity density, 3‐D connectivity index; a measure of the degree to which a structure is multiply connected), and TRI‐SMI (structure model index; is related to the architecture of the structure and ranges between 0 and 3, SMI toward 0 or lower signifies that the structure is mainly concave plates, whereas a value of three means only cylindrical rods).

### Bone histology and histomorphometry

Bone histology and histomorphometry analysis was performed at Yale Core Center for Musculoskeletal Disorders at Yale University School of Medicine (New Haven, CT). The measurements, terminology, and units used were those recommended by the Nomenclature Committee of the American Society for Bone and Mineral Research (Dempster et al. 2013). Femurs were dissected, fixed in 10% neutral buffered formalin for 24 h before transferring to 70% ethanol. Bones were then dehydrated and embedded undecalcified in methyl methacrylate (MMA). Longitudinal sections, 5 *μ*m thick, were cut along the frontal plane from the MMA plastic‐embedded blocks using a Leica 2265 microtome (Microm, Richards‐Allan Scientific, Kalamazoo, MI, USA) and stained with toluidine blue. For dynamic histomorphometric analyses, each mouse received two SC injections of 30 mg/kg body weight calcein at 10 and 3 days before sacrifice. Static parameters of bone formation and resorption were measured at a standardized site in an area of the distal femoral epiphysis spanning from 75 *μ*m from the growth plate to the endocortical edge of the epiphysis, as described previously (Tawfeek et al. 2010) using an Olympus BX40 microscope interfaced with the Osteomeasure system software and hardware (Osteometrics, Atlanta, GA). Software calculations of various parameters are based on previously defined formulas (Dempster et al. 2013). BV/TV (percentage of bone volume relative to total tissue volume), Tb Th (trabecular thickness), Tb N (trabecular number), OV/TV (percentage of osteoid volume relative to total tissue volume), OV/BV (percentage of osteoid volume relative to bone volume), BS/BV (percentage of bone surface relative to bone volume), OS/BS (percentage of osteoid surface relative to total bone surface), O Th (osteoid thickness), Ob.S/BS (percentage of bone surface covered by osteoblasts), N.Ob/BS (osteoblast number per millimeter bone surface), Ob.S/BS (percentage of bone surface covered by osteoblasts), N.Oc/BS (osteoclast number per millimeter bone surface), Oc.S/BS (percentage of bone surface occupied by osteoclasts), N.Ob/B.Pm (osteoblast number per bone perimeter), N. Oc/B.Pm (osteoclast number per bone perimeter), N.Ob/T.Ar (osteoblast number per total tissue area), and N.Oc/T.Ar (osteoclast number per total tissue area) were measured.

### Measurement of serum markers of bone turnover

Blood was collected in BD Microtainer™ Capillary Blood Collector and BD Microgard™ Closure (BD cat. #BD 365967) drawn by cardiac puncture which was performed as a terminal procedure on isoflurane‐anesthetized animals. Sera were then isolated and frozen at −80°C. Serum markers of bone turnover were measured, as described previously (Gao et al. 2008; Terauchi et al. 2009; Tawfeek et al. 2010) and by following the manufacturer's instructions. Serum collagen type I C‐telopeptide or carboxy‐terminal collagen crosslinks (CTX), a marker of bone resorption, procollagen type 1 intact N‐terminal propeptide (P1NP), a specific marker of bone resorption, and tartrate‐resistant acid phosphatase (TRAcP‐5b, a specific serum marker of osteoclastic activity) were measured using rodent‐specific ELISA assay kits from Immunodiagnostic Systems (Scottsdale, AZ).

### Statistical analysis

As per experimental protocol, animals were randomized between sham‐vehicle, SCI‐vehicle, and SCI‐PTH groups and the analyses were performed blindly regarding the control and test groups by using unique animal identification codes. The results are presented as the means ± SD. Unpaired Student's *t*‐test was used for comparisons between two groups. For carrying out multiple comparisons between all groups, one‐way ANOVA and Tukey's post hoc test were employed. Statistical significance was considered when the *P* value was less than 0.05 (*P* < 0.05).

## Results

### PTH improves trabecular bone structure in SCI animals

**Figure 1:**
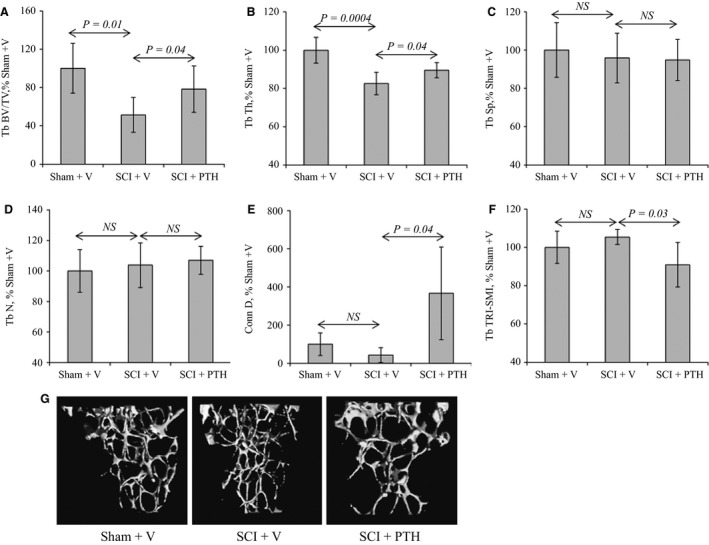
PTH improves trabecular bone structure in SCI mice: Female mice underwent sham or SCI surgery then were injected SC daily, except Sunday, with vehicle or PTH (80 *μ*g/kg/day) for 35 days. Proximal regions of tibias from sham‐vehicle (Sham+V)‐, SCI‐vehicle (SCI+V)‐, and SCI‐PTH (SCI+PTH)‐treated mice were analyzed by micro‐CT. (A) Tb BV/TV is trabecular bone volume/total volume. (B) Tb Th is trabecular thickness. (C) Tb Sp is trabecular spacing or separation. (D) Tb N is trabecular number. (E) Tb Conn D is trabecular connectivity density. (F) Tb TRI SMI is trabecular three‐dimensional structure model index. (G) Images: Examples of proximal tibia micro‐CT scans. The data in all graphs are expressed as the means ± SD. *N* = 6 mice/group.

To assess the effects of intermittent PTH administration on trabecular bone, sham and SCI animals were injected daily (except Sunday) SC with vehicle or PTH for 35 days. Mice were then sacrificed and the isolated tibias were analyzed by micro*‐*CT. SCI‐vehicle animals exhibited a marked decrease in proximal tibial fractional trabecular bone volume (Tb BV/TV) and trabecular thickness (Tb Th) compared to the control sham‐vehicle group (Fig. 1A and B). Additionally, two indices that have been implicated in mechanical properties of bone, connectivity density (Conn D), and structure model index (TRI‐SMI) were examined after SCI. Although there was a trend for a decrease in Conn D with higher TRI‐SMI in the trabecular bone of the SCI‐vehicle compared to sham‐vehicle animals, these differences did not reach significance (Fig. 1E and F).

Interestingly, PTH treatment protected against SCI‐induced trabecular bone loss and preserved bone architecture. SCI animals that received daily PTH injection had significantly higher Tb BV/TV, Tb Th, and Conn D, and lower TRI‐SMI, than SCI‐vehicle counterparts (Fig. 1A, B, E, and F). There was no significant difference in trabecular separation (Tb Sp) or number (Tb N) among the groups (Fig. 1C and D). Qualitative changes in trabecular bone structure are also demonstrated in micro‐CT images of the proximal tibia (Fig. 1G).

### PTH attenuates cortical bone loss and reduces porosity in SCI animals

**Figure 2:**
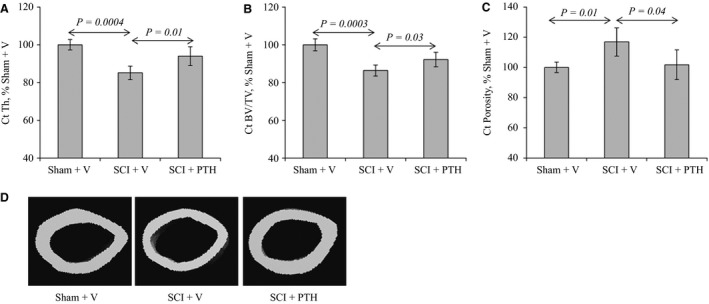
PTH increases cortical bone volume and thickness and reduces porosity in SCI mice: Female mice underwent sham or SCI surgery then were injected SC daily, except Sunday, with vehicle or PTH (80 *μ*g/kg/day) for 35 days. Midshaft regions of femurs from sham‐vehicle (Sham+V)‐, SCI‐vehicle (SCI+V)‐, and SCI‐PTH (SCI+PTH)‐treated mice were analyzed by micro‐CT. (A) Ct Th is cortical thickness. (B) Ct BV/TV is cortical bone volume/total volume. (C) Ct porosity is cortical porosity. (D) Images: Examples of femur midshaft micro‐CT scans. The data in all graph are expressed as the means ± SD. *N* = 6 mice/group.

To determine the effects of PTH treatment on cortical bone structure after SCI, sham‐ and SCI‐operated animals were injected daily with vehicle or PTH for 35 days. Midshaft regions of isolated femurs were then analyzed by micro*‐*CT. Micro‐CT analysis revealed that SCI‐vehicle animals suffered a reduction in cortical bone volume (Ct BV/TV) and thickness (Ct Th), and an elevation in cortical porosity (Ct Porosity) compared to the sham‐vehicle group (Fig. 2A–C). In contrast, PTH injection preserved cortical thickness and volume and abolished the increase in porosity observed in femurs of SCI animals (Fig. 2A–C). Qualitative changes in cortical bone are also demonstrated in micro‐CT images of the femur midshaft region (Fig. 2D).

### PTH prevents the deleterious effects of SCI on histomophometrical indices of bone formation and resorption

To understand further how PTH protects against SCI‐induced bone loss and improves bone structure and microarchitecture, bone histology and histomorphometry were performed on femurs isolated from sham‐vehicle, SCI‐vehicle, and SCI‐PTH animal groups.

**Figure 3:**
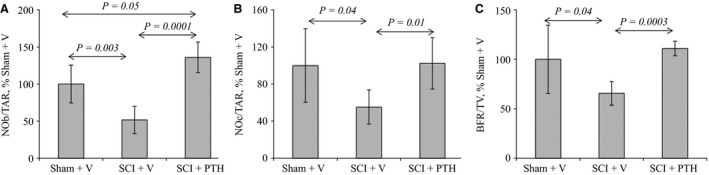
PTH improves static and dynamic histomorphometrical indices of bone formation and resorption: (A) Analysis of static indices of bone formation in distal femur region: Female mice underwent sham or SCI surgery then were injected SC daily, except Sunday, with vehicle or PTH (80 *μ*g/kg/day) for 35 days. Femurs harvested from sham‐vehicle (Sham+V)‐, SCI‐vehicle (SCI+V)‐, and SCI‐PTH (SCI+PTH)‐treated mice were processed as described in the methods sections then stained with toluidine blue and osteoblast numbers were counted. N.Ob/T.Ar is number of osteoblasts per total tissue area. (B) Analysis of static indices of bone resorption in distal femur region: Female mice underwent sham or SCI surgery then were injected SC daily, except Sunday, with vehicle or PTH (80 *μ*g/kg/day) for 35 days. Femurs were harvested from sham‐vehicle (Sham+V)‐, SCI‐vehicle (SCI+V)‐, and SCI‐PTH (SCI+PTH)‐treated mice and processed as described in the methods sections then stained with toluidine blue and osteoclast numbers were counted. N.Oc/T.Ar is the number of osteoclasts per total tissue area. (C) Analysis of dynamic indices of bone formation in femur: Femurs were harvested from female sham‐vehicle (Sham+V)‐, SCI‐vehicle (SCI+V)‐, and SCI‐PTH (SCI+PTH)‐treated mice and bone formation rate (BFR) was evaluated using calcein labeling. The data in all graphs are expressed as the means ± SD. *N* = 6 mice/group.

To assess the effects of daily PTHR1 activation on osteoblast and osteoclast numbers, static histomorphometry analysis was performed. The results show that two important static indices of bone formation (N.Ob/T.Ar) and bone resorption (N.Oc/T.Ar) that were considerably reduced in SCI‐vehicle animals were augmented as a result of PTH treatment (Fig. 3A and B). Furthermore, dynamic bone histomorphometry evaluation demonstrated that SCI animals had pronounced reduction in bone formation rate (BFR) compared to sham‐vehicle control (Fig. 3C). This reduction in BFR was completely prevented by PTH treatment of SCI animals and BFR became similar to sham‐vehicle controls (Fig. 3C).

Consistently, other indices, namely BV/TV, OV/TV, Tb Sp, Tb N, Ob.S/OS, and N.Ob/Opm were also significantly increased in the SCI‐PTH animals compared to SCI‐vehicle and/or sham‐vehicle controls (Table 1). Other histomorphometrical parameters were not significantly altered (Table 1).

**Table 1:** Bone histomorphometry analysis of distal femur region from female sham‐vehicle (Sham+V), SCI‐vehicle (SCI+V), and SCI‐PTH (SCI+PTH) mouse groups**.**

Bone parameter	Sham+V mean ± SD	SCI+V mean ± SD (*P* value vs. Sham+V*)*	SCI+PTH mean ± SD (*P* value vs. SCI+V*)*
BV/TV	9 ± 2	5 ± 1 (0.008)	11 ± 2 (0.0005)
Tb Sp	468 ± 141	782 ± 112 (0.01)	358 ± 86 (0.0004)
Tb N	2.1 ± 0.5	1.3 ± 0.2 (0.009)	2.7 ± 0.5 (0.001)
O Th	3.7 ± 0.2	3.4 ± 0.2 (NS)	3.5 ± 0.2 (NS)
Tb Th	34 ± 12	37 ± 4 (NS)	39 ± 3 (NS)
OV/TV	0.9 ± 0.2	0.5 ± 0.1 (0.01)	1 ± 0.2 (0.001)
OV/BV	9.7 ± 1.5	10 ± 2 (NS)	10 ± 0.6 (NS)
BS/BV	48 ± 2.3	54 ± 6.5 (NS)	50 ± 5 (NS)
OS/BS	55 ± 4.5	53 ± 9 (NS)	54 ± 6 (NS)
Ob.S/BS	37 ± 5	33 ± 11 (NS)	40 ± 5 (NS)
Ob.S/OS	68 ± 3	59 ± 11 (NS)	73 ± 1 (0.03)
Oc.S/BS	5.2 ± 1	5.8 ± 2 (NS)	4.2 ± 0.8 (NS)
N.Ob/B.Pm	37 ± 5	32 ± 9 (NS)	41 ± 5 (NS)
N.Ob/Opm	68 ± 4	60 ± 8 (NS)	76 ± 4 (0.01)
N.Ob/Obpm	100 ± 3	100 ± 6 (NS)	103 ± 4 (NS)
N.Oc/B.Pm	1.6 ± 0.4	1.6 ± 0.6 (NS)	1.3 ± 0.3 (NS)

Results are presented as means ± SD. *N* = 6 mice/group. Parameters are defined under the methods section. NS is not significant.

### PTH promotes the serum levels of bone turnover markers

**Figure 4:**
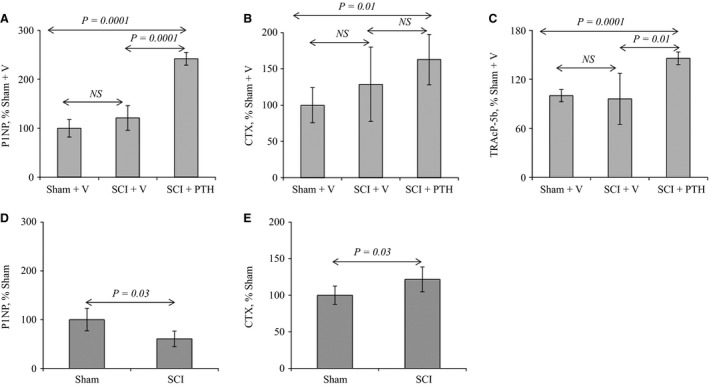
Changes in the serum levels of bone turnover markers after SCI and PTH treatment: Upper panel: PTH increases the serum levels of bone turnover markers in SCI mice: Female mice underwent sham or SCI surgery then were injected SC daily, except Sunday, with vehicle or PTH (80 *μ*g/kg/day) for 35 days. Sera were collected from sham‐vehicle (Sham+V)‐, SCI‐vehicle (SCI+V)‐, and SCI‐PTH (SCI+PTH)‐treated mice. Bone formation and resorption markers were determined by measuring serum P1NP, TRAcP‐5b and CTX, respectively, by ELISA. (A) Serum levels of P1NP. (B) Serum levels of CTX. (C) Serum levels of TRAcP‐5b. Lower panel: Serum levels of bone turnover markers are altered after 14 days of SCI: Female mice underwent sham or SCI surgery and sera were collected after 14 days and levels of P1NP and CTX were measured by ELISA. (D) Serum levels of P1NP. (E) Serum levels of CTX. The data in all graphs are expressed as the means ± SD. *N* = 5–6 mice/group.

To determine the effects of PTH treatment on serum markers of bone turnover after SCI, markers of bone formation and resorption were measured by ELISA. Analysis of sera collected from sham‐vehicle and SCI‐vehicle mice showed no significant changes in either bone formation (Procollagen I intact N‐terminal propeptide, P1NP) or resorption (collagen type 1 cross‐linked C‐telopeptide, CTX, and osteoclastic activity‐specific tartrate‐resistant acid phosphatase, TRAcP‐5b) markers between sham‐vehicle and SCI‐vehicle control animals (Fig. 4A–C). PTH administration, however, caused greater stimulation of bone turnover in SCI animals. PTH caused an elevation of serum P1NP, CTX, and TRAcP‐5b to levels that were significantly higher than sham‐vehicle and SCI‐vehicle groups (Fig. 4A–C). These results suggest that PTHR1 activation promotes bone turnover in SCI animals.

To determine if serum levels of bone turnover are altered in the early stage of SCI, animals underwent sham and SCI surgery for 14 days. Sera were collected and P1NP and CTX levels were measured by ELISA. The results show that P1NP levels were significantly lower while CTX were higher in SCI than sham control animals after 14 days (Fig. 4D and E).

## Discussion

The high incidence and severity of sublesional osteoporosis after SCI and the absence of effective conservative (rehabilitation) or pharmacological therapeutic regimen has made bone loss in this condition a difficult clinical challenge to address (Maimoun et al. 2006a). There is, therefore, a pressing need for additional and focused research to explore new therapeutic approaches that would reduce fracture risk in patients with SCI.

In this report, using a mouse model, we demonstrate that severe immobilization, as a result of SCI, is associated with marked attenuation of bone turnover, causing severe cortical and trabecular bone loss in the distal femoral and proximal tibial regions. We further show, for the first time, that daily PTH administration protects against SCI‐induced bone loss by promoting both bone formation and resorption, thus maintaining bone microarchitecture.

Our static histomorphometry findings demonstrated that both osteoclast and osteoblast cell numbers were evidently reduced in bones of the paralyzed hindlimbs after 35 days. Dynamic histomorphomerty analysis further revealed that bone formation rate was attenuated in immobilized animals. These results support the conclusion that inhibition of bone turnover and uncoupling of bone resorption and formation contributes to bone loss after SCI. These results may explain, at least partly, why patients with osteoporosis after SCI fail to respond to antiresorptive agents that act principally by inhibiting osteoclast formation and activity (Bauman et al. 2014). Of relevance, osteocytic osteolysis and osteocyte‐induced bone degradation and demineralization, a concept that was raised decades ago, has recently been reintroduced as a possible mechanism for bone dissolution and osteolysis in many forms of bone loss including immobilization (Baylink et al. 1973; Tazawa et al. 2004; Teti and Zallone 2009; Qing et al. 2012; Wysolmerski 2012, 2013; Blaber et al. 2013; Lloyd et al. 2014; Clarke et al. 2015; Nango et al. 2016). Our finding of increased cortical porositiy implies that osteocyte osteolysis could partially account for bone demineralization and loss after SCI. However, further investigation is warranted to verify whether or not osteocytic osteolysis is involved in rapid bone dissolution after SCI.

As reported previously (Maimoun et al. 2002, 2005; Zehnder et al. 2004; Kostovski et al. 2015), when assessed earlier (14 days), P1NP levels were lower and CTX were higher in SCI than sham control animals. However, at 35 days, despite the clear reduction in the local indices of bone formation and resorption in SCI animals, there was no significant change in the serum levels of bone turnover markers. Collectively, these results suggest that bone loss after SCI is likely initiated by activation of bone resorption before osteoblast lineage and osteoclast cell functions are severely compromised at later stages of SCI; this leads to a status of low bone turnover and subsequent adynamic bone disease. Notably, absence of concomitant decline in the circulating bone makers after 35 days of SCI may be attributed to masking by possible rise in bone turnover subsequent to adaptation to weight bearing on the intact forelimb bones.

With the advancement of medical care, patients with SCI have near‐normal longevity, and, with the benefit of technological advances, they are increasingly upright and active. Moreover, powered exoskeletal devices place increased forces on the sublesional long‐bones of the lower extremities, which must be strong enough to withstand the added stresses and strains without fracturing. The development of pharmacological approaches that reduce bone loss and retain bone strength would be anticipated to prevent fractures, with associated improved quality of life. With the current uncertainty surrounding the use of antiresorptive agents and other rehabilitation measures to prevent bone loss after SCI, bone anabolic agents emerge as an attractive approach that deserves serious attention. In contrast to the antiresorptive agents that act by preventing further bone loss, bone anabolic agents act primarily on osteoblasts to stimulate bone formation and improve bone mass, structure, and mechanical properties. Thus, assessing the existing food and drug administration (FDA)‐approved bone anabolic drugs offers hope for preventing bone loss in patients with SCI.

In this regard, human PTH(1–34) or teriparatide, the amino terminal fragment of PTH, is a proven, effective bone anabolic agent and has been approved by the FDA for treating both postmenopausal (Neer et al. 2001) and glucocorticoid induced (Saag et al. 2007) osteoporosis. In these patients, PTH increases bone mineral density (BMD) at the spine and femur of osteoporotic men and women, and decreases by greater than half the risk of vertebral and nonvertebral fractures. PTH is also relatively safe and is superior to other FDA‐approved therapies for osteoporosis (e.g., antiresorptive agents) in improving bone density and quality, and reducing fracture risk.

Surprisingly, there has been relatively insufficient focus on evaluating PTH as a potential therapy for bone loss after SCI in animal models or human studies. There is only one recent, and quite limited, pilot study in man that administered PTH to subjects with SCI. The study had serious limitations and was inconclusive, principally due to the lack of a control group, short duration of treatment (6–12 months of PTH), and small sample size (11 subjects received 6 months of PTH and 7 subjects received 12 months of PTH). Moreover, the subjects were studied 3–21 years after SCI, with a mean duration of injury of approximately 10 years (only 2 subjects were injured less than 5 years—that is, after significant bone loss had already occurred, and the sublesional skeleton was probably in a low turnover state for the majority of subjects studied (Gordon et al. 2013). Regarding the animal studies, rat and mouse models of skeletal unloading have been utilized to assess the effects of PTHR1 activation on disuse osteopenia (Ma et al. 1995; Halloran et al. 1997; Turner et al. 1998, 2007; Ono et al. 2007; Bruel et al. 2013; Sandberg et al. 2014). In these studies, hindlimb unloading was induced pharmacologically using botulin toxin A (Botox) (Bruel et al. 2013; Sandberg et al. 2014) or mechanically using tail suspension or limb fixation to the abdomen (Ma et al. 1995; Halloran et al. 1997; Turner et al. 1998, 2007; Ono et al. 2007). These investigations have demonstrated that PTH under different experimental conditions namely wide dose range (1–200 *μ*g/kg/day), short‐ or long‐term treatment duration, or daily or continuous administration efficiently counteracts trabecular and cortical bone loss in the unloaded femur and tibia. PTH was further shown to reduce bone loss primarily by enhancing bone formation, but by also reducing osteoclast number in some of these studies.

Accordingly and in light of the urgent demand for effective therapy to reduce bone loss after SCI, we evaluated the efficacy of PTH in reducing bone loss after SCI. Our findings reveal that intermittent PTHR1 activation is a potentially appealing strategy for treatment of bone loss in patients with nonambulatory SCI. Our biochemical and histological analyses strongly demonstrate that PTHR1 activation triggers a strong bone turnover response in immobilized animals. The micro‐CT analysis further indicates that the increase in bone turnover positively impacts cortical bone thickness and porosity, enhances trabecular bone volume and architecture, and increases trabecular connectivity and the number of concave plate‐ over rod‐like structures. A decrease in the plate/rod ratio and connectivity are believed to be major contributing factors to fragility fracture in various forms of osteoporosis and with advancing age, while an increase in these parameters induced by treatment with bone anabolic and anti‐resorptive agents is beneficial for bone quality and strength (Ding and Hvid 2000; Benito et al. 2003; Borah et al. 2004; Rupprecht et al. 2006; Benhamou 2007; Liu et al. 2013; Altman et al. 2014; Stein et al. 2014; Sutter et al. 2014; Zhou et al. 2014; Chang et al. 2015). These effects of PTH on bone formation and bone structure and architecture are consistent with those reported under other hindlimb unloading conditions (Ma et al. 1995; Halloran et al. 1997; Turner et al. 1998, 2007; Ono et al. 2007; Bruel et al. 2013; Sandberg et al. 2014). However, PTH stimulation of both bone formation and resorption has only been observed in SCI further underscoring the differential impact of SCI on osteoblast lineage and osteoclast cell function and their responsiveness to PTH.

Lastly, it is important to acknowledge the limitations of the present investigation. First, while the course of PTH treatment started immediately after SCI, further investigations are required to address the efficacy of PTH after chronic SCI. Second, while we anticipate that PTH will be efficacious in male animals, this should be independently verified.

In conclusion, the present study proposes that intermittent PTHR1 activation is an effective anabolic approach that should be seriously evaluated for restoring bone integrity in patients with severe disuse osteoporosis.

## Conflict of Interest

The authors declare no competing financial interests.

## References

[phy213446-bib-0001] Altman, A. R. , W. J. Tseng , C. M. de Bakker , B. K. Huh , A. Chandra , L. Qin , et al. 2014 A closer look at the immediate trabecula response to combined parathyroid hormone and alendronate treatment. Bone 61:149–157. 24468717 10.1016/j.bone.2014.01.008 PMC3972893

[phy213446-bib-0002] Battaglino, R. A. , A. A. Lazzari , E. Garshick , and L. R. Morse . 2012 Spinal cord injury‐induced osteoporosis: pathogenesis and emerging therapies. Curr. Osteoporos. Rep. 10:278–285. 22983921 10.1007/s11914-012-0117-0 PMC3508135

[phy213446-bib-0003] Bauman, W. A. , and C. P. Cardozo . 2015 Osteoporosis in individuals with spinal cord injury. PM R 7:188–201; quiz 201, 2015. 25171878 10.1016/j.pmrj.2014.08.948

[phy213446-bib-0004] Bauman, W. A. , C. M. Cirnigliaro , M. F. La Fountaine , L. Martinez , S. C. Kirshblum , and A. M. Spungen . 2014 Zoledronic acid administration failed to prevent bone loss at the knee in persons with acute spinal cord injury: an observational cohort study. J. Bone Miner. Metab. 33:410–421. 25158630 10.1007/s00774-014-0602-x

[phy213446-bib-0005] Baylink, D. , J. Sipe , J. Wergedal , and O. J. Whittemore . 1973 Vitamin D‐enhanced osteocytic and osteoclastic bone resorption. Am. J. Physiol. 224:1345–1357. 4712149 10.1152/ajplegacy.1973.224.6.1345

[phy213446-bib-0006] Bedi, B. , J. Y. Li , H. Tawfeek , K. H. Baek , J. Adams , S. S. Vangara , et al. 2012 Silencing of parathyroid hormone (PTH) receptor 1 in T cells blunts the bone anabolic activity of PTH. Proc. Natl Acad. Sci. USA 109:E725–E733. 22393015 10.1073/pnas.1120735109 PMC3311391

[phy213446-bib-0007] Benhamou, C. L. 2007 Effects of osteoporosis medications on bone quality. Joint Bone Spine 74:39–47. 17196423 10.1016/j.jbspin.2006.06.004

[phy213446-bib-0008] Benito, M. , B. Gomberg , F. W. Wehrli , R. H. Weening , B. Zemel , A. C. Wright , et al. 2003 Deterioration of trabecular architecture in hypogonadal men. J. Clin. Endocrinol. Metab. 88:1497–1502. 12679429 10.1210/jc.2002-021429

[phy213446-bib-0009] Blaber, E. A. , N. Dvorochkin , C. Lee , J. S. Alwood , R. Yousuf , P. Pianetta , et al. 2013 Microgravity induces pelvic bone loss through osteoclastic activity, osteocytic osteolysis, and osteoblastic cell cycle inhibition by CDKN1a/p21. PLoS ONE 8:e61372. 23637819 10.1371/journal.pone.0061372 PMC3630201

[phy213446-bib-0010] Borah, B. , T. E. Dufresne , P. A. Chmielewski , T. D. Johnson , A. Chines , and M. D. Manhart . 2004 Risedronate preserves bone architecture in postmenopausal women with osteoporosis as measured by three‐dimensional microcomputed tomography. Bone 34:736–746. 15050906 10.1016/j.bone.2003.12.013

[phy213446-bib-0011] Bouxsein, M. L. , S. K. Boyd , B. A. Christiansen , R. E. Guldberg , K. J. Jepsen , and R. Muller . 2010 Guidelines for assessment of bone microstructure in rodents using micro‐computed tomography. J. Bone Miner. Res. 25:1468–1486. 20533309 10.1002/jbmr.141

[phy213446-bib-0012] Bruel, A. , J. B. Vegger , A. C. Raffalt , J. E. Andersen , and J. S. Thomsen . 2013 PTH (1‐34), but not strontium ranelate counteract loss of trabecular thickness and bone strength in disuse osteopenic rats. Bone 53:51–58. 23246791 10.1016/j.bone.2012.11.037

[phy213446-bib-0013] Chang, G. , S. Honig , Y. Liu , C. Chen , K. K. Chu , C. S. Rajapakse , et al. 2015 7 Tesla MRI of bone microarchitecture discriminates between women without and with fragility fractures who do not differ by bone mineral density. J. Bone Miner. Metab. 33:285–293. 24752823 10.1007/s00774-014-0588-4 PMC4363287

[phy213446-bib-0014] Clarke, M. V. , P. K. Russell , D. M. Findlay , S. Sastra , P. H. Anderson , J. P. Skinner , et al. 2015 A role for the calcitonin receptor to limit bone loss during lactation in female mice by inhibiting osteocytic osteolysis. Endocrinology 156:3203–3214. 26135836 10.1210/en.2015-1345

[phy213446-bib-0015] Dauty, M. , B. Perrouin Verbe , Y. Maugars , C. Dubois , and J. F. Mathe . 2000 Supralesional and sublesional bone mineral density in spinal cord‐injured patients. Bone 27:305–309. 10913927 10.1016/s8756-3282(00)00326-4

[phy213446-bib-0016] Dempster, D. W. , J. E. Compston , M. K. Drezner , F. H. Glorieux , J. A. Kanis , H. Malluche , et al. 2013 Standardized nomenclature, symbols, and units for bone histomorphometry: a 2012 update of the report of the ASBMR Histomorphometry Nomenclature Committee. J. Bone Miner. Res. 28:2–17. 23197339 10.1002/jbmr.1805 PMC3672237

[phy213446-bib-0017] Demulder, A. , M. Guns , A. Ismail , E. Wilmet , P. Fondu , and P. Bergmann . 1998 Increased osteoclast‐like cells formation in long‐term bone marrow cultures from patients with a spinal cord injury. Calcif. Tissue Int. 63:396–400. 9799824 10.1007/s002239900547

[phy213446-bib-0018] Ding, M. , and I. Hvid . 2000 Quantification of age‐related changes in the structure model type and trabecular thickness of human tibial cancellous bone. Bone 26:291–295. 10710004 10.1016/s8756-3282(99)00281-1

[phy213446-bib-0019] Gao, Y. , X. Wu , M. Terauchi , J. Y. Li , F. Grassi , S. Galley , et al. 2008 T cells potentiate PTH‐induced cortical bone loss through CD40L signaling. Cell Metab. 8:132–145. 18680714 10.1016/j.cmet.2008.07.001 PMC2569843

[phy213446-bib-0020] Gaspar, A. P. , C. M. Brandao , and M. Lazaretti‐Castro . 2014 Bone mass and hormone analysis in spinal cord injury patients: evidences for a gonadal axis disruption. J. Clin. Endocrinol. Metab. 99:4649–4655. 25222754 10.1210/jc.2014-2165

[phy213446-bib-0021] Gordon, K. E. , M. J. Wald , and T. J. Schnitzer . 2013 Effect of parathyroid hormone combined with gait training on bone density and bone architecture in people with chronic spinal cord injury. PM R 5:663–671. 23558091 10.1016/j.pmrj.2013.03.032

[phy213446-bib-0022] Halloran, B. P. , D. D. Bikle , J. Harris , S. Tanner , T. Curren , and E. Morey‐Holton . 1997 Regional responsiveness of the tibia to intermittent administration of parathyroid hormone as affected by skeletal unloading. J. Bone Miner. Res. 12:1068–1074. 9200006 10.1359/jbmr.1997.12.7.1068

[phy213446-bib-0023] Huang, H. F. , M. T. Li , T. A. Linsenmeyer , J. E. Ottenweller , L. M. Pogach , and R. J. Irwin . 1997 The effects of spinal cord injury on the status of messenger ribonucleic acid for TRPM 2 and androgen receptor in the prostate of the rat. J. Androl. 18:250–256. 9203052

[phy213446-bib-0024] Jiang, S. D. , L. Y. Dai , and L. S. Jiang . 2006 Osteoporosis after spinal cord injury. Osteoporos. Int. 17:180–192. 16217589 10.1007/s00198-005-2028-8

[phy213446-bib-0025] Jiang, S. D. , L. S. Jiang , and L. Y. Dai . 2007 Effects of spinal cord injury on osteoblastogenesis, osteoclastogenesis and gene expression profiling in osteoblasts in young rats. Osteoporos. Int. 18:339–349. 17036173 10.1007/s00198-006-0229-4

[phy213446-bib-0026] Kostovski, E. , P. O. Iversen , and N. Hjeltnes . 2010 Complications of chronic spinal cord injury. Tidsskr. Nor. Laegeforen. 130:1242–1245. 20567276 10.4045/tidsskr.09.0055

[phy213446-bib-0027] Kostovski, E. , N. Hjeltnes , E. F. Eriksen , S. O. Kolset , and P. O. Iversen . 2015 Differences in bone mineral density, markers of bone turnover and extracellular matrix and daily life muscular activity among patients with recent motor‐incomplete versus motor‐complete spinal cord injury. Calcif. Tissue Int. 96:145–154. 25539858 10.1007/s00223-014-9947-3

[phy213446-bib-0028] Liu, X. S. , J. Wang , B. Zhou , E. Stein , X. Shi , M. Adams , et al. 2013 Fast trabecular bone strength predictions of HR‐pQCT and individual trabeculae segmentation‐based plate and rod finite element model discriminate postmenopausal vertebral fractures. J. Bone Miner. Res. 28:1666–1678. 23456922 10.1002/jbmr.1919 PMC3688669

[phy213446-bib-0029] Lloyd, S. A. , A. E. Loiselle , Y. Zhang , and H. J. Donahue . 2014 Evidence for the role of connexin 43‐mediated intercellular communication in the process of intracortical bone resorption via osteocytic osteolysis. BMC Musculoskelet. Disord. 15:122. 24716486 10.1186/1471-2474-15-122 PMC3984635

[phy213446-bib-0030] Ma, Y. F. , W. S. Jee , H. Z. Ke , B. Y. Lin , X. G. Liang , M. Li , et al. 1995 Human parathyroid hormone‐(1‐38) restores cancellous bone to the immobilized, osteopenic proximal tibial metaphysis in rats. J. Bone Miner. Res. 10:496–505. 7785472 10.1002/jbmr.5650100322

[phy213446-bib-0031] Maimoun, L. , I. Couret , J. P. Micallef , E. Peruchon , D. Mariano‐Goulart , M. Rossi , et al. 2002 Use of bone biochemical markers with dual‐energy X‐ray absorptiometry for early determination of bone loss in persons with spinal cord injury. Metabolism 51:958–963. 12145766 10.1053/meta.2002.34013

[phy213446-bib-0032] Maimoun, L. , I. Couret , D. Mariano‐Goulart , A. M. Dupuy , J. P. Micallef , E. Peruchon , et al. 2005 Changes in osteoprotegerin/RANKL system, bone mineral density, and bone biochemicals markers in patients with recent spinal cord injury. Calcif. Tissue Int. 76:404–411. 15812577 10.1007/s00223-004-0048-6

[phy213446-bib-0033] Maimoun, L. , C. Fattal , J. P. Micallef , E. Peruchon , and P. Rabischong . 2006a Bone loss in spinal cord‐injured patients: from physiopathology to therapy. Spinal Cord 44:203–210. 16158075 10.1038/sj.sc.3101832

[phy213446-bib-0034] Maimoun, L. , S. Lumbroso , F. Paris , I. Couret , E. Peruchon , E. Rouays‐Mabit , et al. 2006b The role of androgens or growth factors in the bone resorption process in recent spinal cord injured patients: a cross‐sectional study. Spinal Cord 44:791–797. 16568142 10.1038/sj.sc.3101922

[phy213446-bib-0035] Morse, L. , Y. D. Teng , L. Pham , K. Newton , D. Yu , W. L. Liao , et al. 2008 Spinal cord injury causes rapid osteoclastic resorption and growth plate abnormalities in growing rats (SCI‐induced bone loss in growing rats). Osteoporos. Int. 19:645–652. 17987335 10.1007/s00198-007-0494-x PMC4370281

[phy213446-bib-0036] Morse, L. R. , Y. Xu , B. Solomon , L. Boyle , S. Yoganathan , P. Stashenko , et al. 2011 Severe spinal cord injury causes immediate multi‐cellular dysfunction at the chondro‐osseous junction. Transl. Stroke Res. 2:643–650. 22368723 10.1007/s12975-011-0118-9 PMC3285243

[phy213446-bib-0037] Nance, P. W. , O. Schryvers , W. Leslie , S. Ludwig , J. Krahn , and D. Uebelhart . 1999 Intravenous pamidronate attenuates bone density loss after acute spinal cord injury. Arch. Phys. Med. Rehabil. 80:243–251. 10084430 10.1016/s0003-9993(99)90133-8

[phy213446-bib-0038] Nango, N. , S. Kubota , T. Hasegawa , W. Yashiro , A. Momose , and K. Matsuo . 2016 Osteocyte‐directed bone demineralization along canaliculi. Bone 84:279–288. 26709236 10.1016/j.bone.2015.12.006

[phy213446-bib-0039] Neer, R. M. , C. D. Arnaud , J. R. Zanchetta , R. Prince , G. A. Gaich , J. Y. Reginster , et al. 2001 Effect of parathyroid hormone (1‐34) on fractures and bone mineral density in postmenopausal women with osteoporosis. N. Engl. J. Med. 344:1434–1441. 11346808 10.1056/NEJM200105103441904

[phy213446-bib-0040] Ono, N. , K. Nakashima , E. Schipani , T. Hayata , Y. Ezura , K. Soma , et al. 2007 Constitutively active parathyroid hormone receptor signaling in cells in osteoblastic lineage suppresses mechanical unloading‐induced bone resorption. J. Biol. Chem. 282:25509–25516. 17500070 10.1074/jbc.M610782200 PMC3595314

[phy213446-bib-0041] Qin, W. , W. A. Bauman , and C. P. Cardozo . 2010 Evolving concepts in neurogenic osteoporosis. Curr. Osteoporos. Rep. 8:212–218. 20820963 10.1007/s11914-010-0029-9

[phy213446-bib-0042] Qing, H. , L. Ardeshirpour , P. D. Pajevic , V. Dusevich , K. Jahn , S. Kato , et al. 2012 Demonstration of osteocytic perilacunar/canalicular remodeling in mice during lactation. J. Bone Miner. Res. 27:1018–1029. 22308018 10.1002/jbmr.1567 PMC3770147

[phy213446-bib-0043] Reiter, A. L. , A. Volk , J. Vollmar , B. Fromm , and H. J. Gerner . 2007 Changes of basic bone turnover parameters in short‐term and long‐term patients with spinal cord injury. Eur. Spine J. 16:771–776. 16830131 10.1007/s00586-006-0163-3 PMC2200720

[phy213446-bib-0044] Roberts, D. , W. Lee , R. C. Cuneo , J. Wittmann , G. Ward , R. Flatman , et al. 1998 Longitudinal study of bone turnover after acute spinal cord injury. J. Clin. Endocrinol. Metab. 83:415–422. 9467550 10.1210/jcem.83.2.4581

[phy213446-bib-0045] Rupprecht, M. , P. Pogoda , M. Mumme , J. M. Rueger , K. Puschel , and M. Amling . 2006 Bone microarchitecture of the calcaneus and its changes in aging: a histomorphometric analysis of 60 human specimens. J. Orthop. Res. 24:664–674. 16514636 10.1002/jor.20099

[phy213446-bib-0046] Saag, K. G. , E. Shane , S. Boonen , F. Marin , D. W. Donley , K. A. Taylor , et al. 2007 Teriparatide or alendronate in glucocorticoid‐induced osteoporosis. N. Engl. J. Med. 357:2028–2039. 18003959 10.1056/NEJMoa071408

[phy213446-bib-0047] Sabour, H. , A. Norouzi Javidan , S. Latifi , B. Larijani , F. Shidfar , M. R. Vafa , et al. 2014 Bone biomarkers in patients with chronic traumatic spinal cord injury. Spine J. 14:1132–1138. 24139865 10.1016/j.spinee.2013.07.475

[phy213446-bib-0048] Sandberg, O. , B. R. Macias , and P. Aspenberg . 2014 Low dose PTH improves metaphyseal bone healing more when muscles are paralyzed. Bone 63:15–19. 24582802 10.1016/j.bone.2014.02.008

[phy213446-bib-0049] Stein, E. M. , A. Kepley , M. Walker , T. L. Nickolas , K. Nishiyama , B. Zhou , et al. 2014 Skeletal structure in postmenopausal women with osteopenia and fractures is characterized by abnormal trabecular plates and cortical thinning. J. Bone Miner. Res. 29:1101–1109. 24877245 10.1002/jbmr.2144 PMC4084559

[phy213446-bib-0050] Sutter, S. , K. K. Nishiyama , A. Kepley , B. Zhou , J. Wang , D. J. McMahon , et al. 2014 Abnormalities in cortical bone, trabecular plates, and stiffness in postmenopausal women treated with glucocorticoids. J. Clin. Endocrinol. Metab. 99:4231–4240. 25127089 10.1210/jc.2014-2177 PMC4223438

[phy213446-bib-0051] Tawfeek, H. , B. Bedi , J. Y. Li , J. Adams , T. Kobayashi , M. N. Weitzmann , et al. 2010 Disruption of PTH receptor 1 in T cells protects against PTH‐induced bone loss. PLoS ONE 5:e12290. 20808842 10.1371/journal.pone.0012290 PMC2924900

[phy213446-bib-0052] Tazawa, K. , K. Hoshi , S. Kawamoto , M. Tanaka , S. Ejiri , and H. Ozawa . 2004 Osteocytic osteolysis observed in rats to which parathyroid hormone was continuously administered. J. Bone Miner. Metab. 22:524–529. 15490261 10.1007/s00774-004-0519-x

[phy213446-bib-0053] Terauchi, M. , J. Y. Li , B. Bedi , K. H. Baek , H. Tawfeek , S. Galley , et al. 2009 T lymphocytes amplify the anabolic activity of parathyroid hormone through Wnt10b signaling. Cell Metab. 10:229–240. 19723499 10.1016/j.cmet.2009.07.010 PMC2751855

[phy213446-bib-0054] Teti, A. , and A. Zallone . 2009 Do osteocytes contribute to bone mineral homeostasis? Osteocytic osteolysis revisited Bone 44:11–16. 18977320 10.1016/j.bone.2008.09.017

[phy213446-bib-0055] Turner, R. T. , G. L. Evans , J. M. Cavolina , B. Halloran , and E. Morey‐Holton . 1998 Programmed administration of parathyroid hormone increases bone formation and reduces bone loss in hindlimb‐unloaded ovariectomized rats. Endocrinology 139:4086–4091. 9751486 10.1210/endo.139.10.6227

[phy213446-bib-0056] Turner, R. T. , G. L. Evans , S. Lotinun , P. D. Lapke , U. T. Iwaniec , and E. Morey‐Holton . 2007 Dose‐response effects of intermittent PTH on cancellous bone in hindlimb unloaded rats. J. Bone Miner. Res. 22:64–71. 17042715 10.1359/jbmr.061006

[phy213446-bib-0057] Uebelhart, D. , D. Hartmann , H. Vuagnat , M. Castanier , H. J. Hachen , and A. Chantraine . 1994 Early modifications of biochemical markers of bone metabolism in spinal cord injury patients. A preliminary study. Scand. J. Rehabil. Med. 26:197–202. 7878394

[phy213446-bib-0058] Wysolmerski, J. J. 2012 Osteocytic osteolysis: time for a second look? Bonekey Rep. 1:229. 24363929 10.1038/bonekey.2012.229 PMC3868715

[phy213446-bib-0059] Wysolmerski, J. J. 2013 Osteocytes remove and replace perilacunar mineral during reproductive cycles. Bone 54:230–236. 23352996 10.1016/j.bone.2013.01.025 PMC3624069

[phy213446-bib-0060] Zehnder, Y. , M. Luthi , D. Michel , H. Knecht , R. Perrelet , I. Neto , et al. 2004 Long‐term changes in bone metabolism, bone mineral density, quantitative ultrasound parameters, and fracture incidence after spinal cord injury: a cross‐sectional observational study in 100 paraplegic men. Osteoporos. Int. 15:180–189. 14722626 10.1007/s00198-003-1529-6

[phy213446-bib-0061] Zhou, B. , X. S. Liu , J. Wang , X. L. Lu , A. J. Fields , and X. E. Guo . 2014 Dependence of mechanical properties of trabecular bone on plate‐rod microstructure determined by individual trabecula segmentation (ITS). J. Biomech. 47:702–708. 24360196 10.1016/j.jbiomech.2013.11.039 PMC3925244

